# Efficacy of a Novel Tapered Contrast Catheter for Endoscopic Ultrasound-Guided Hepaticogastrostomy: A Multicenter Study

**DOI:** 10.3390/jcm13061580

**Published:** 2024-03-10

**Authors:** Fumitaka Niiya, Tatsunori Sato, Junichi Kaneko, Kazuma Ishikawa, Naoki Tamai, Masataka Yamawaki, Jun Noda, Tetsushi Azami, Fumiya Nishimoto, Yuichi Takano, Masatsugu Nagahama

**Affiliations:** 1 Division of Gastroenterology, Showa University Fujigaoka Hospital, Yokohama 227-8501, Japan; tama.226st.hope@gmail.com (N.T.); masatakaymwk@outlook.jp (M.Y.); nodaji0317@gmail.com (J.N.); azamitetsushidesu@gmail.com (T.A.); nishifumi0815@gmail.com (F.N.); yuichitakano1028@yahoo.co.jp (Y.T.); masatsugu.nagahama@gmail.com (M.N.); 2 Department of Gastroenterology, Shizuoka General Hospital, Shizuoka 420-0881, Japan; tatsunorisato@i.shizuokapho.jp; 3 Division of Gastroenterology, Iwata City Hospital, Iwata 438-0002, Japan; meganerock10@gmail.com; 4 Department of Medical Oncology, School of Medicine, Sapporo Medical University, Sapporo 060-8543, Japan; naganuma2002@gmail.com

**Keywords:** endoscopic ultrasound-guided hepaticogastrostomy, endoscopic retrograde cholangiopancreatography, retrospective study, contrast catheter, novel

## Abstract

**Background**: Endoscopic ultrasound-guided hepaticogastrostomy (EUS-HGS) is an alternative for failed endoscopic retrograde cholangiopancreatography (ERCP), with current success rates of 65–84% considered suboptimal. A novel ERCP catheter (SHOREN, Kaneka Corporation, Osaka, Japan) with a tapered 3.3-French tip may facilitate smoother insertion, potentially improving outcomes. **Methods**: This retrospective analysis encompassed EUS-HGS procedures conducted from January 2021 to August 2023 at four institutions. The aim of this study was to compare the performance of conventional and novel ERCP contrast catheters regarding the success rate of single-attempt catheter insertion, failure rates, technical success rates, and incidence of adverse events. **Results**: The study included 48 patients; 26 underwent EUS-HGS using conventional catheters and 22 with the novel catheter. The novel catheter achieved higher success rates in single-attempt insertions (96.5% vs. 80.8%) and lower failure rates (4.6% vs. 7.7%). The occurrence of bile peritonitis was comparable between the two groups. **Conclusions**: The novel ERCP contrast catheter with a tapered tip appears to contribute to successful catheter insertion and is useful for EUS-HGS.

## 1. Introduction

Endoscopic retrograde cholangiopancreatography (ERCP) is the standard procedure for the treatment of malignant biliary obstruction [1,2]. However, ERCP has the potential to fail because of complications such as duodenal obstruction and surgically altered anatomy. Percutaneous transhepatic biliary drainage (PTBD) has previously been performed as an alternative to ERCP; however, it has a high rate of adverse events (AEs) [3]. Recently, Endoscopic ultrasound-guided biliary drainage (EUS-BD) has been widely performed instead of PTBD, and the efficacy of endoscopic ultrasound-guided hepaticogastrostomy (EUS-HGS) has been described in previous reports [4,5,6]. Although a high success rate of EUS-HGS has been reported [7], comprehensive identification and analysis of risk factors for AEs remain an area of ongoing research. Bile leakage is a severe AE of EUS-HGS that can occur during and after the procedure, with an incidence ranging from 2.8% to 11% [8,9]. This AE can be fatal; therefore, an effective method to reduce bile leakage is desirable. The usefulness of bile aspiration after the insertion of an ERCP contrast catheter has been reported recently [10]. This method is acceptable because increased bile pressure can be a risk factor for cholangitis after ERCP. ERCP contrast catheters are widely used for bile aspiration before dilation; however, the type of catheter that is preferable for EUS-HGS is unknown. The success rate of catheter insertion has been reported to be 65–84%, which might be considered suboptimal [11,12]. Therefore, an ERCP contrast catheter that can be easily inserted into the bile duct is highly desirable.

Recently, a novel ERCP contrast catheter (SHOREN; Kaneka Corporation, Osaka, Japan) that has a tapered 3.3-French (Fr) tip was developed. The tapered tip of this catheter may be useful for a smooth insertion during this procedure. No studies have been conducted on the efficacy of this novel ERCP catheter for EUS-HGS. Therefore, the aim of this study was to compare the conventional ERCP contrast catheter with the novel ERCP contrast catheter and evaluate its efficacy and safety.

## 2. Methods

### 2.1. Ethics Statements

This study was approved by the local institutional review board (approval number: 2023-115-A) and performed in accordance with the principles of the Declaration of Helsinki.

### 2.2. Study Design and Population

**Figure 1:**
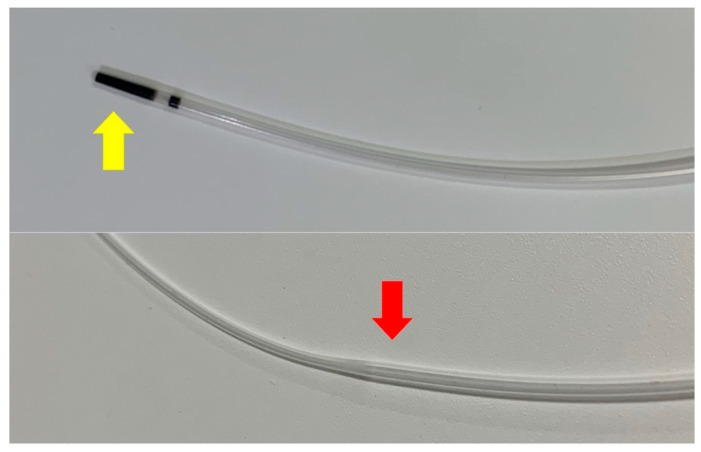
Image of the novel ERCP contrast catheter. This device has a 3.3-Fr tapered tip (yellow arrowhead) and a 5.9-Fr wider lumen (red arrowhead). Almost no gaps are observed at the top of the catheter. ERCP, endoscopic retrograde cholangiopancreatography; Fr, French.

We retrospectively analyzed consecutive patients who underwent EUS-HGS between January 2021 and August 2023 at four institutions. We excluded patients who met the following criteria: (1) the bile duct was punctured from B2 during EUS-HGS, (2) an ERCP contrast catheter was not used, (3) the bile duct puncture failed, and (4) there were insufficient data. Figure 1 shows the novel tapered ERCP contrast catheter (SHOREN; Kaneka Corporation, Tokyo, Japan) used for EUS-HGS in this study. This catheter has a tapered 3.3-Fr tip and a wide lumen to aspirate bile juice easily. On the contrary, a conventional ERCP contrast catheter (MTW Endoskopie, Düsseldorf, Germany) with a 4.9-Fr tip was used for EUS-HGS.

### 2.3. EUS-HGS

Endoscopists with previous experience of >20 EUS-HGSs performed all EUS-HGS procedures. All the procedures were performed using a conventional curved linear array echoendoscope (GF-UCT260; Olympus Medical Systems, Tokyo, Japan). An endoscopic ultrasound (EUS) scope was inserted into the stomach. After the left intrahepatic bile duct was detected on endoscopic ultrasonography, it was punctured using a 19-gauge (G) EUS-fine needle aspiration needle. The bile duct was identified by injecting a contrast medium, and a 0.025-inch guidewire (VisiGlide2, Olympus Medical Systems) was advanced into the bile duct.

A conventional ERCP contrast catheter (MTW Endoskopie, Düsseldorf, Germany) or novel ERCP contrast catheter (SHOREN, Tokyo, Japan) was inserted over the guidewire, and the bile was aspirated. If the ERCP contrast catheter could not be inserted into the bile duct, it was inserted after tract dilation using dilation devices (bougie, balloon, or cautery catheters). Following tract dilation, a covered metallic stent (8 mm in diameter and 12 cm in length, bare-end type, Niti-S biliary S-type; Tae-Woong Corporation, Seoul, Korea; 8 mm in diameter and 12 cm in length, Spring Stopper Stent; Tae-Woong Corporation) or a 7-Fr plastic stent (Through-Pass type IT, Gadelius Medical, Tokyo, Japan) was deployed from the intrahepatic duct into the stomach. The decision on the type of ERCP contrast catheter was made by each endoscopist. More than 10 mL of bile was aspirated in all procedures.

### 2.4. Outcome Measurements

**Figure 2:**
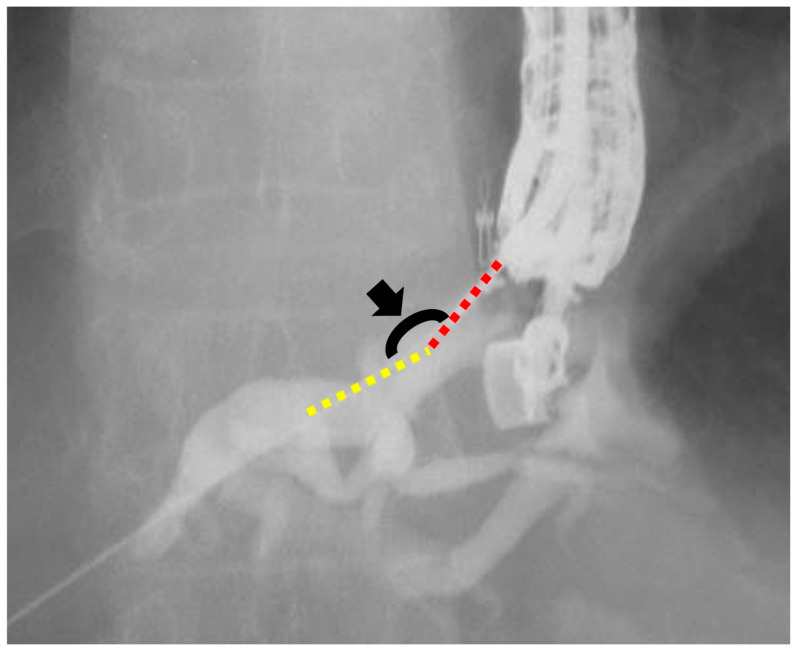
Measurement of the insertion angle. The insertion angle (black arrowhead) is defined as the angle between the guidewire in the liver parenchyma (yellow dotted line) and the punctured peripheral intrahepatic bile duct (red dotted line).

We defined the conventional and novel groups as patients who underwent EUS-HGS using a conventional ERCP contrast catheter (MTW Endoskopie) or the novel ERCP contrast catheter (SHOREN), respectively. Patient data were collected from the electronic medical records and endoscopy databases. We compared outcomes between the conventional and novel groups. The primary outcome was the success rate of ERCP contrast catheter insertion in a single attempt. Secondary outcomes were the failure rate of ERCP contrast catheter insertion, insertion guidewire angle, overall technical success rate, procedure time, and AEs. These outcomes were compared between the two groups. Successful ERCP contrast catheter insertion was defined as the successful insertion of the ERCP contrast catheter into the intrahepatic duct. Successful ERCP contrast catheter insertion in a single attempt was defined as successful on the first attempt. If we pulled the ERCP catheter after the first attempt of catheter insertion or adjusted the scope position, ERCP catheter insertion in a single attempt was considered a failure. The insertion angle was defined as the angle between the guidewire in the liver parenchyma and the peripheral intrahepatic bile duct that was punctured (Figure 2). The overall technical success was defined as stent deployment in the intended position. The procedure time was measured from endoscope insertion to stent deployment. The AEs were defined and graded according to the American Society for Gastrointestinal Endoscopy Severity Grading system [13]. Bile peritonitis was diagnosed on the basis of ultrasonography or computed tomography findings and was defined as the presence of fluid collection after EUS-HGS, in addition to symptoms of infection, such as elevated infection markers on blood tests. Fever was defined as a fever of >38 °C persisting for 24 h after the EUS-HGS procedure. Abdominal pain was defined as new-onset abdominal pain that worsened after the EUS-HGS procedure.

### 2.5. Statistical Analysis

Continuous variables are reported as medians and interquartile ranges. Categorical variables are presented as proportions.

## 3. Results

### 3.1. Patient Characteristics

During the study period, 90 patients underwent EUS-HGS. After excluding 42 EUS-HGS cases (the bile duct was punctured from B2 during EUS-HGS, n = 21; an ERCP contrast catheter was not used, n = 17; the bile duct puncture failed, n = 1; insufficient data, n = 3), 48 EUS-HGS cases were included in this study (conventional group, n = 26; novel group, n = 22).

Patient characteristics are shown in Table 1. There were 26 patients (mean age, 76 years; 15 males) in the conventional group and 22 patients (mean age, 76.5 years; 10 males) in the novel group. Regarding disease, pancreatic cancer was the most common primary disease in both groups. The site of biliary obstruction was mostly distal obstruction in both groups.

### 3.2. Characteristics of the Procedure

Table 2 presents the characteristics of the procedure. The most frequent puncture site was the intrahepatic bile duct of the III segment (B3) in both groups. There were no differences in the median diameter of the intrahepatic bile duct between the groups (conventional group, 6 mm; novel group, 5 mm). All procedures were performed using a 19G puncture needle. Regarding the hepaticogastrostomy stent type, metal stents were mostly used, and antegrade stenting was performed in two cases in each group. The median procedure time was 29 min and 25.5 min in the conventional and novel groups, respectively.

### 3.3. Outcomes

The study outcomes are presented in Table 3. The success rates of ERCP catheter insertion in a single attempt were 80.8% (21/26) in the conventional group and 96.5% (21/22) in the novel group. The median guidewire angles were 114° and 90° in the conventional and novel groups, respectively. The failure rates of ERCP catheter insertion were 7.7% (2/26) and 4.6% (1/22) in the conventional and novel groups, respectively. The technical success rate was 100% in each group.

### 3.4. AEs

Table 4 summarizes the AEs. Bile peritonitis was observed in three cases of the conventional group and in four cases of the novel group. Sepsis occurred in one patient in the novel group. Other AEs included cholangitis in one case of the conventional group, bleeding in one case of the novel group, and acute pancreatitis and abdominal pain in one case of the conventional group.

## 4. Discussion

In this study, we compared the outcomes of EUS-HGS using a conventional ERCP contrast catheter with those of a novel ERCP contrast catheter. The novel ERCP catheter, with its 3.3-Fr tapered tip, facilitated easier insertion into the intrahepatic bile duct during EUS-HGS. This is the first study to evaluate the efficacy of the novel ERCP catheter during EUS-HGS.

Bile peritonitis after EUS-HGS can be fatal. Therefore, methods to prevent the AEs that may occur at each step of EUS-HGS have been suggested. Recently, one-step EUS-HGS, a method of inserting a metal stent without dilation, has been reported [13,14]. This is an ideal technique to reduce bile leakage; however, there is a risk of deploying the stent outside the bile duct because it is sometimes difficult to confirm the bile duct without a sufficient contrast medium injection. From this perspective, ERCP contrast catheters, allowing steady detection of the bile duct with contrast media, were widely used for EUS-HGS. Another reason for the use of an ERCP contrast catheter is bile aspiration. Previous studies have reported that bile aspiration prior to track dilation reduces AEs [10,11]; this is another ideal solution for the prevention of bile leaks. However, the insertion of an ERCP catheter is technically challenging because of the puncture site and insertion guidewire angle, and few studies have reported the type of catheter that is suitable for EUS-HGS.

The novel ERCP catheter has a more tapered tip (3.3-Fr) than a conventional ERCP catheter, which may be helpful for smooth insertion of the catheter into the bile duct. In the present study, we defined the level of ease for insertion of an ERCP contrast catheter as the success rate of ERCP contrast catheter insertion in a single attempt. The success rates of ERCP catheter insertion in a single attempt were 96.5% in the novel group and 80.8% in the conventional group. Fujii et al. reported that a wide angle at the insertion site was an independent predictor of successful cannula insertion [12], and the success rate of ERCP contrast catheter insertion in a single attempt using a novel ERCP contrast catheter was higher than that using a conventional ERCP contrast catheter, even though the guidewire angle in the novel group was narrower than that in the conventional group. These findings suggest that the novel ERCP contrast catheter may be more suitable than a conventional ERCP contrast catheter in terms of ease of insertion.

Previously, most reports have recommended that EUS-HGS be performed in tertiary care and experienced centers [15,16]. Hara et al. suggested that endoscopists perform at least the first 20 cases with an expert endoscopist of EUS-HGS [17] because there is an observed learning curve for EUS-HGS. In this study, all endoscopists performed >20 EUS-HGS procedures, which might have contributed to the higher success rate of ERCP contrast catheter insertion than in previous reports. If the endoscopist is a trainee in EUS-HGS, this new ERCP contrast catheter would be more effective for the procedure than the conventional contrast catheter. Currently, this procedure is gradually being performed in general hospitals with the generalization of this technique. Taken together, in this case, a more sophisticated device, such as a tapered catheter, may be useful during the procedure.

The overall complication rate of EUS-HGS (25%, 12/48) in this study was within the range reported previously. We expected a lower frequency of bile peritonitis in the novel group than in the conventional group because of the wider catheter lumen, which may enable bile aspiration easily. However, in this study, no significant differences were observed between the groups. We believe that one of the reasons for this unexpected result was not evaluating factors for AEs, e.g., the amount of aspirated bile. Further studies are required to examine the effectiveness of this device in larger populations. However, we believe that this novel ERCP contrast catheter with a wider lumen can be helpful in reducing bile leakage because bile aspiration has been identified as significantly effective in preventing AEs.

This study had several limitations. First, some bias exists owing to the small sample size and retrospective study design. Moreover, we excluded EUS-HGS cases punctured from B2 because it is easier to insert devices than at other puncture sites, which may have caused selection bias. Second, in terms of AEs, the fact that the amount of bile aspirated is unknown is the reason for the difficulty in elucidating the factors associated with bile peritonitis. However, this novel ERCP contrast catheter has a wider lumen than a conventional ERCP catheter, which may be effective in reducing AEs. Third, all procedures were performed by an experienced endoscopist, which could have affected the outcomes of catheter insertion. However, if a trainee endoscopist were included in this study, the difference in the rate of ERCP contrast catheter insertion could have widened.

In conclusion, it demonstrates an advantage in smooth insertion due to its tapered tip, despite a narrower guidewire angle, making it suitable for EUS intervention procedures.

Large-scale studies comparing this novel ERCP catheter with a conventional ERCP catheter are required to determine the most effective and safest treatment approaches for EUS-HGS.

## Figures and Tables

**Table 1:** Patients’ characteristics.

		Conventional Group n = 26	Novel Group n = 22
Age, median (IQR), years		76 (67.8–82)	76.5 (70–76.5)
Male sex, n		15	10
Disease, n			
	Pancreatic cancer	12	13
	Cholangiocarcinoma	8	5
	Other type of cancer	3	3
	Benign stricture	3	1
Site of biliary obstruction, n			
	Distal	18	16
	Hilar	7	6
	Hepaticojejunostomy	1	0
Ascites, n		6	3
Malignant duodenum obstruction, n		17	7

IQR, interquartile range.

**Table 2:** Characteristics of the procedure.

		Conventional Group n = 26	Novel Group n = 22
Puncture site, n			
	B3	26	21
	Site other than B3	0	1
Intrahepatic bile duct diameter, median (IQR), mm		6 (4–7)	5 (4–6)
HGS stent type, n			
	Metal	19	13
	Plastic	7	9
Antegrade stenting, n		2	2
Procedure time, median (IQR), minutes		29 (22–39)	25.5 (19–36.5)

IQR, interquartile range; HGS, hepaticogastrostomy.

**Table 3:** Outcome measures.

	Conventional Group n = 26	Novel Group n = 22
Success rate of ERCP contrast catheter insertion in one attempt, % (n)	80.8 (21/26)	96.5 (21/22)
Insertion guidewire angle, median (IQR), °	114 (90–138)	90 (85–107.5)
Failure rate of ERCP contrast catheter insertion, % (n)	7.7 (2/26)	4.6 (1/22)
Technical success rate, % (n)	100 (26/26)	100 (22/22)

IQR, interquartile range; ERCP, endoscopic retrograde cholangiopancreatography.

**Table 4:** Adverse events.

		Conventional Group n = 26	Novel Group n = 22
Total, n (%)		6 (23.1)	6 (27.2)
	Bile peritonitis	3	4
	Sepsis	0	1
	Cholangitis	1	0
	Bleeding	0	1
	Acute pancreatitis	1	0
	Abdominal pain	1	0

## Data Availability

Dataset available on request from the authors.

## Abbreviations

ERCP: endoscopic retrograde cholangiopancreatography; PTBD: percutaneous transhepatic biliary drainage; AEs: adverse events; EUS-BD: endoscopic ultrasound-guided biliary drainage; EUS-HGS: endoscopic ultrasound-guided hepaticogastrostomy; Fr: French; EUS: endoscopic ultrasound; G: gauge.
